# The Simultaneous Effects of miR-145-5p and hsa-let-7a-3p on Colorectal Tumorigenesis: In Vitro Evidence

**DOI:** 10.34172/apb.2024.004

**Published:** 2023-07-19

**Authors:** Nazila Mozammel, Elham Baghbani, Mohammad Amini, Sheyda Jodeiry Zaer, Yalda Baghay Esfandyari, Maryam Tohidast, Seyed Samad Hosseini, Seyed Ali Rahmani, Ahad Mokhtarzadeh, Behzad Baradaran

**Affiliations:** ^1^Department of Biology, Higher Education Institute of Rab‐Rashid, Tabriz, Iran.; ^2^Immunology Research Center, Tabriz University of Medical Sciences, Tabriz, Iran.

**Keywords:** Colorectal cancer, MIR-145-5p, hsa-let-7a-3p

## Abstract

**Purpose:**

MicroRNAs (miRNAs) are a group of small regulatory non-coding RNAs, which are dysregulated through tumor progression. let-7 and MIR-145 are both tumor suppressor microRNAs that are downregulated in a wide array of cancers including colorectal cancer (CRC).

**Methods:**

This study was aimed to investigate the effect of simultaneous replacement of these two tumor suppressor miRNAs on proliferation, apoptosis, and migration of CRC cells. HCT-116 with lower expression levels of hsa-let-7a-3p and MIR-145-5p was selected for functional investigations. The cells were cultured and transfected with hsa-let-7a and MIR-145, separately and in combination. Cell viability and apoptosis rates were assessed by MTT assay and flow cytometry, respectively. Cell cycle status was further evaluated using flow cytometry and qRT-PCR was employed to evaluate gene expression.

**Results:**

The obtained results showed that exogenous overexpression of MIR-145 and hsa-let-7a in HCT-116 cells could cooperatively decrease CRC cell proliferation and induce sub-G1 cell cycle arrest. Moreover, hsa-let-7a and MIR-145 co-transfection significantly increased apoptosis induction compared to separate transfected cells and control through modulating the expression levels of apoptosis-related genes including *Bax*, *Bcl-2*, *P53*, *Caspase-3*, *Caspase-8*, and *Caspase-9*. Furthermore, qRT-PCR results illustrated that hsa-let-7a and MIR-145 combination more effectively downregulated *MMP-9* and *MMP-2* expression, as the important modulators of metastasis, compared to the controls.

**Conclusion:**

Taken together, considering that exogenous overexpression of MIR-145 and hsa-let-7a showed cooperative anti-cancer effects on CRC cells, their combination may be considered as a novel therapeutic strategy for the treatment of CRC.

## Introduction

 Colorectal cancer (CRC) is one the most frequent form of human cancer, with a high rate of metastasis and invasion that causes thousands of deaths worldwide, making CRC the second fatal malignancy.^1^ In 2018, around 1.8 million new cases and 881 000 deaths were recorded,^2,3^ young patients suffering from CRC are increasing.^4^ It affects both men and women equally. CRC is a heterogeneous disease, and its risk factors may include lifestyle, diet, obesity, physical inactivity, smoking, stress, and ethnic genetic and epigenetic variations.^5,6^ Despite the recent advances, due to resistance to chemotherapy and the development of metastatic diseases after tumor resection, as the main treatment options, CRC largely remains an incurable malignancy. Hence, exploring the molecular mechanisms that drive CRC tumorigenesis and metastasis could offer valuable insights for advancing innovative therapeutic strategies to enhance CRC treatment.^7^ The non-coding RNA (ncRNA) molecules were identified as essential regulatory factors that may function as tumor suppressors or oncogenes in human cancers. Their abnormal expression dysregulates many biological pathways through cancer pathogenesis.^8-10^ Increasing evidence has demonstrated that ncRNAs, particularly microRNAs (miRNAs), possess high potential as diagnostic and therapeutic targets for better managing human malignancies.^11-14^ miRNAs are small, single-stranded, endogenous ncRNA molecules with a length of approximately 19–22 nucleotides.^15^ The special features are low abundance and high sequence homology between family members.^16^ miRNAs exhibit efficacy as post-transcriptional regulators, suppressing gene expression by binding to their target mRNAs’ 3’-untranslated regions (3′‐UTR) and subsequently influencing various biological processes.^17^ Recent studies have illustrated that miRNAs are dysregulated in human cancers, including CRC, and play crucial roles in tumorigenesis, metastasis, invasion, and drug resistance,^18^ introducing them as valuable targets for cancer therapy.^19^ As the well-established gene therapy methods, miRNAs are recruited in miRNA replacement therapy. One therapeutic approach involves restoring tumor suppressor miRNA mimics through transfection, aiming to restore their function and explore the therapeutic potential of miRNAs. In particular, MIR-145 and let-7 are remarkably downregulated in various cancers, including breast, gastric, lung, and CRC, and function as the tumor suppressor miRNAs through inhibition of tumor growth, metastasis, and chemoresistance, possessing great potential as therapeutic targets.^20^

 The MIR-145 gene is located in the 1.6 kb region of chromosome 5q33.1, a conserved location in the genome, with 4.08 kb length, which is often deleted through tumorigenesis.^21^ It was shown that MIR-145 regulates cell apoptosis, proliferation, and differentiation of stem cells.^22^ In various human cancers, this tumor suppressor miRNA is commonly downregulated and influences carcinogenesis via targeting multiple genes and pathways.^23^ MIR-145 was reported to be downregulated in CRC primary tumors, which was correlated with the aggressive form of malignancy. Overexpression of MIR-145 was further demonstrated to inhibit CRC cell growth, migration, and invasion.^24,25^ Furthermore, let-7, another promising tumor suppressor miRNA involved in human cancer progression,^26^ is also downregulated during CRC tumorigenesis, and its reduced expression contributes to CRC cell proliferation, migration, and stemness.^27^

 In this study, given the critical role of these two miRNAs in CRC, for the first time, we tried to combine these two miRNAs as a novel cancer gene therapy approach in CRC. Our results demonstrated that MIR-145 and hsa-let-7a were cooperatively able to reduce cell viability through apoptosis induction and induce sub-G1 cell cycle arrest. Furthermore, their combination more effectively downregulated metastasis-related genes, including *MMP-2* and *MMP-9*, suggesting MIR-145 and hsa-let-7a combination presents a hopeful therapeutic approach to the treatment of CRC.

## Materials and Methods

###  Cell culture and cell line selection 

 CRC cell lines, including HT-29 (ATCC; HTB-38), HCT-116 (ATCC; CCL-247), and SW-480 (ATCC; CCL-228), were purchased from the Pasteur Institute of Iran. The cells were grown in RPMI-1640 medium (Gibco) enriched with 10% fetal bovine serum (FBS) and 1% penicillin/streptomycin (Sigma). Incubation of the cells occurred at 37 °C in a humidified environment with 5% CO_2_. They were passaged at 70%-80% confluency and used in all experiments in the logarithmic phase of growth. To select the appropriate cell line, the expression levels of hsa-let-7a and MIR-145 in HCT-116, HT-29, and SW-480 cell lines were evaluated by qRT-PCR, which will be explained in the following.

###  Transfection of miRNAs 

 According to the qRT-PCR results, HCT-116 with the lowest expression levels of hsa-let-7a and MIR-145 was used for functional investigations. The cell precipitate was first dissolved in the electroporation buffer, then 1 × 10^6^ transferred to 500 mL cuvette, then MIR-145-5p and hsa-let-7a-3p (in different doses including 10, 20, and 40 pmol) were added. Gene Pulser Xcell electroporation (BioRad) was employed to transfect miRNA. The cells were incubated for 48 h to determine the optimal dose. 2 × 10^5^/well of transfected cells (with optimal dose) were seeded in 6-well plates and incubated for different times (24, 48, and 72 hours) to select the optimal incubation time. Afterward, using qRT-PCR, the optimum dose and time of miRNAs were determined for further transfection. Furthermore, to assess the transfection efficiency, the cells were transfected with control miRNA conjugated with FITC and visualized using Flow Cytometry (Miltenyi Biotec).

###  RNA extraction, cDNA synthesis and qRT‐PCR

 The Trizol RNA extraction kit (GeneAll, Korea) was utilized for RNA extraction, following the provided protocols. The concentration and purity of the extracted RNA were assessed using the NanoDrop spectrophotometer by measuring the absorbance at A260/A280 (Thermo Scientific, USA). The integrity of RNA was assessed on %1 agarose gel by electrophoresis of total RNA. To determine the expression levels of hsa-let-7a and MIR-145, 1 microgram of RNA was utilized to synthesize complementary DNA (cDNA) employing the Universal cDNA Synthesis miRCURY LNATM kit. Additionally, equal quantities of total RNA were converted into cDNA using the BioFact RT-PCR Pre Mix Synthesis Kit (South Korea) to evaluate the expression levels of the target genes. Real-time PCR was then employed to determine the expression levels of *Bcl-2*, *Bax*, *P53*, *Caspase-3*, *Caspase-8*, *Caspase-9*, *MMP-9*, *MMP-2*, hsa-let-7a, and MIR-145. *U6* and *GAPDH* served as internal controls for normalizing the expression of miRNA and target genes, respectively. The oligonucleotide sequences are displayed in Table 1. Each reaction was conducted independently three times, and the relative expression levels of genes were quantified using the 2^−ΔΔCt^ method.

**Table 1:** List of primer instead oligonucleotide sequences

**Name**	**Forward and reverse**	**Primer sequence**
*Bcl2*	F	5′‐GAGCGTCAACAGGGAGATGTC‐3′
	R	5′‐TGCCGGTTCAGGTACTCAGTC‐3′
*Bax*	F	5′‐TTTGCTTCAGGGTTTCATCCA‐3′
	R	5′‐CTCCATGTTACTGTCCAGTTCGT‐3′
*P53*	F	5′- TTGCAATAGGTGTGCGTCAGA-3′
	R	5′- AGTGCAGGCCAACTTGTTCAG-3′
*Caspase- 8*	F	5′-GGTCTGAAGGCTGGTTGTTC-3′
	R	5′-AATCTCAATATTCCCAAGGTTCAAG-3′
*Caspase- 9*	F	5′-CCGGAATCCTGCTTGGGTATC-3′
	R	5′-CATCGGTGCATTTGGCATGTA-3′
*Caspase-3*	F	5′-TGTCATCTCGCTCTGGTACG-3′
	R	5′-AAATGACCCCTTCATCACCA-3′
*MMP-9*	F	5′-GGTTCTTCTGCGCTACTGCTG-3′
	R	5′-GTCGTAGGGCTGCTGGAAGG-3′
*MMP-2*	F	5′-GGCCTTGCAACCTTGGTCTCTTC-3′
	R	5′-CTCCCTGTGTCAGACTGCTCTTT-3′
*GAPDH*	F	5′- CAAGATCATCAGCAATGCC-3′
	R	5′- GCCATCACGCCACAGTTTCC-3′
*U6*	F	5′-CTTCGGCAGCACATATACTAAAATTGG-3′
	R	5′-TCATCCTTGCGCAGGGG-3′
hsa-let-7a-3p	Target sequence	5′-UGAGGUAGUAGGUUGUAUAGUU-3′
MIR-145-5p	Target sequence	5′-GUCCAGUUUUCCCAGGAAUCCCU-3′

###  MTT assay 

 MTT assay was employed to determine the cell viability and proliferation rates. HCT-116 cells (1 × 10^6^ cells/500 mL) were transfected with hsa-let-7a and MIR-145, in combination or separately, next, 1 × 10^4^ cells were distributed into individual wells of a 96-well plate, followed by an incubation period of 48 hours. The transfected cells with miR-control were used as the negative control (NC). Then, 50 μL of 3-(4, 5- dimethyl thiazolyl-2)-2, 5-diphenyltetrazolium bromide (MTT, 2 mg/mL) solution were added to the wells, and cells were further incubated for 4 hours. Using DMSO, formazan crystals were dissolved, and then UV-visible absorbance was read at the wavelength of 570nm using the 96-well plate reader (Tecan, Switzerland). All reactions were performed in triplicate.

###  Annexin V/propidium iodide (PI) apoptosis assay

 To evaluate apoptosis induction, HCT-116 cells were transfected with MIR-145 and hsa-let-7a mimics, then 2 × 10^5^ cells per well of cells were seeded into 6-well plates. After 48-hour incubation, cells were trypsinized, harvested, and rinsed with PBS. Then, cells were treated with annexin V (5 μL) and propidium iodide (5 μL) in annexin V binding buffer (100 μL) for 15 minutes in a dark. Subsequently, apoptosis induction was investigated by MACS Quant Flow Cytometry. Data were analyzed using FlowJo software.

###  Cell cycle analysis

 To analyze cell cycle progression through treatment groups, HCT-116 cells were transfected with hsa-let-7a or MIR-145 mimics, separately or in combination, and 2 × 10^5^ cells of transfected cells were cultured into each well of a 6-well plate. After 48 hours of incubation, the cells were harvested, rinsed with cold PBS, and fixed in 70% ethanol (1 mL). After overnight incubation at -20° C in ethanol, the cells were rewashed and suspended in 500 mL PBS. Next, 5 μL RNase A was added and incubated for 30 minutes. The cells were then subjected to a washing step, followed by staining with a DAPI solution containing 0.1% Triton X100 and 0.1% DAPI. Subsequently, the cells were incubated in the dark for 30 minutes. The cell cycle status was then evaluated using flow cytometry, and the acquired data were analyzed using FlowJo software.

###  Statistical analysis

 The experimental results were expressed as the mean ± standard deviation. Statistical analysis was conducted using GraphPad Prism 8.0 Software. The student’s t-test was utilized to assess statistical variances between two groups, while comparisons among multiple groups were analyzed using one-way analysis of variance (ANOVA). A *P* value below 0.05 was considered statistically signific

## Results and Discussion

###  Cell line selection 

**Figure 1:**
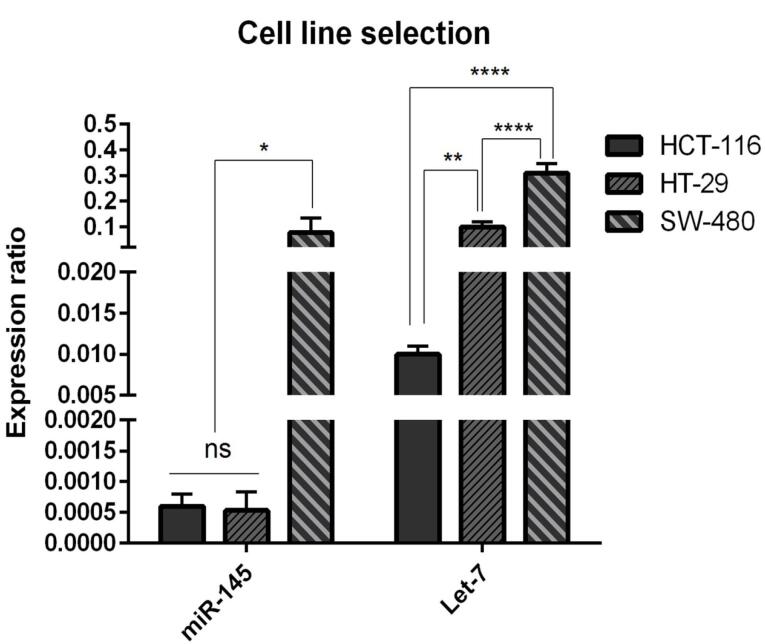
miR‐145 and Let-7 expression levels were evaluated in HTC‐116, HT‐29, SW‐480 CRC cell lines relative to each other. Data are indicated as the mean ± standard deviation of experiments(triplicate); ns = nonsignificant, (* *P* < 0.05), (** *P* < 0.01) and (**** *P* < 0.0001)

 We used qRT-PCR to assess the expression levels of hsa-let-7a and MIR-145 in human CRC cell lines, including SW480, HCT-116, and HT-29. As shown in Figure 1, the expression level of MIR-145 in HCT-116 and HT-29 cell lines was the same (nonsignificant (ns)) but significantly (*P* < 0.05) lower than that of SW-480 cells. Furthermore, hsa-let-7a was remarkably downregulated in HCT-116 cell line in comparison with HT-29 cell line (*P* < 0.01) and SW-480 cell line (*P* < 0.0001). Considering these results, HCT-116 was selected as the proper cell line for further investigations.

###  Transfection efficiency 

**Figure 2:**
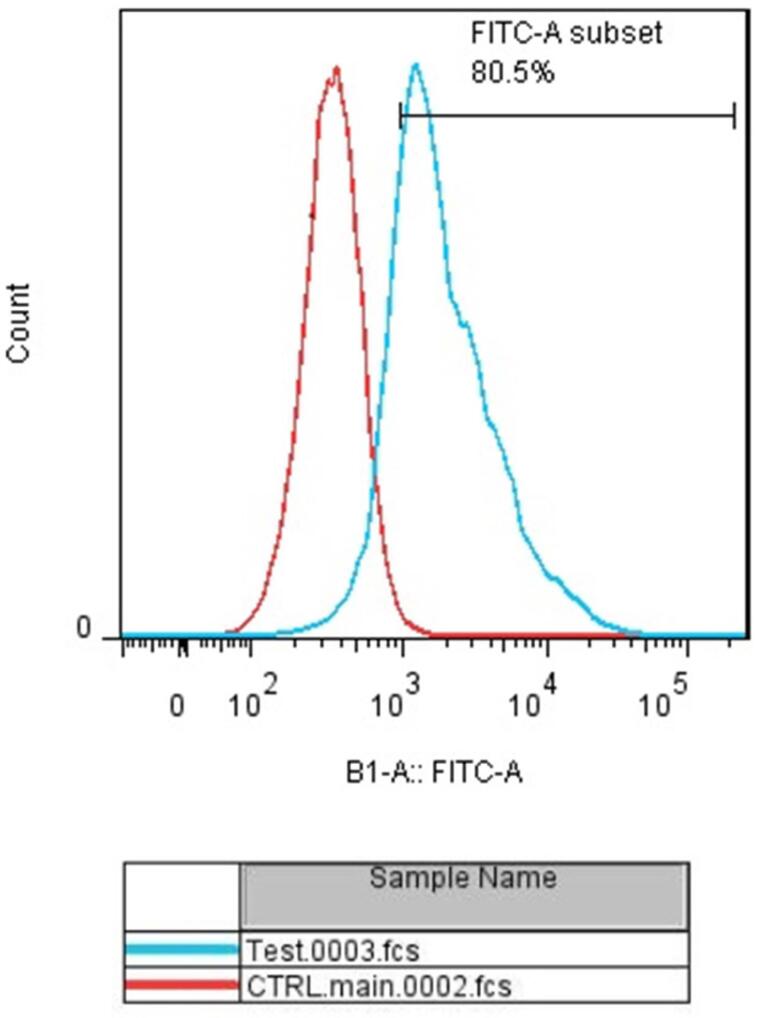
miR‐145 and Let-7a transfection into the HCT‐116 cell line. The efficiency of transfection was evaluated using flow‐cytometry. The transfection rate was estimated at 80.5%

**Figure 3:**
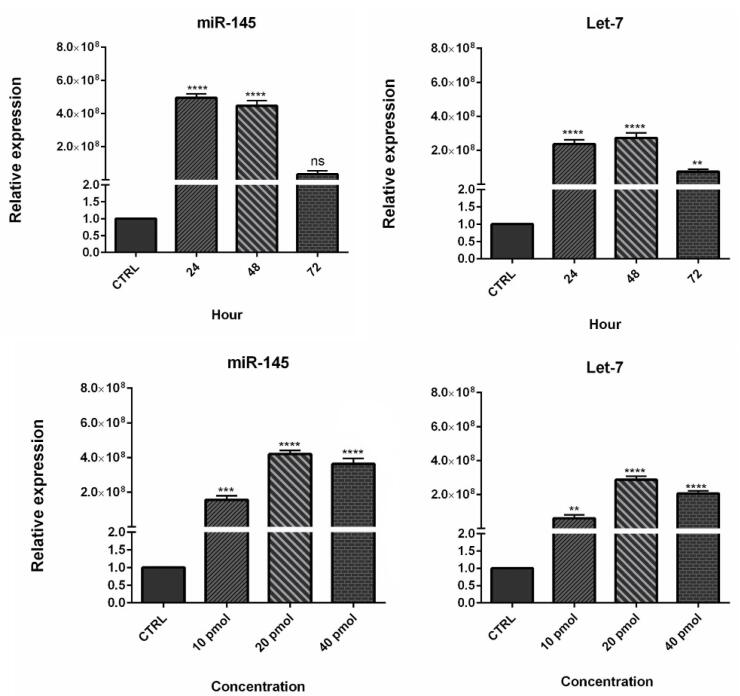
Determination of optimum dose and time for miR-145 and Let-7a. (** *P* < 0.01), (*** *P* < 0.001), and (**** *P* < 0.0001)

**Figure 4:**
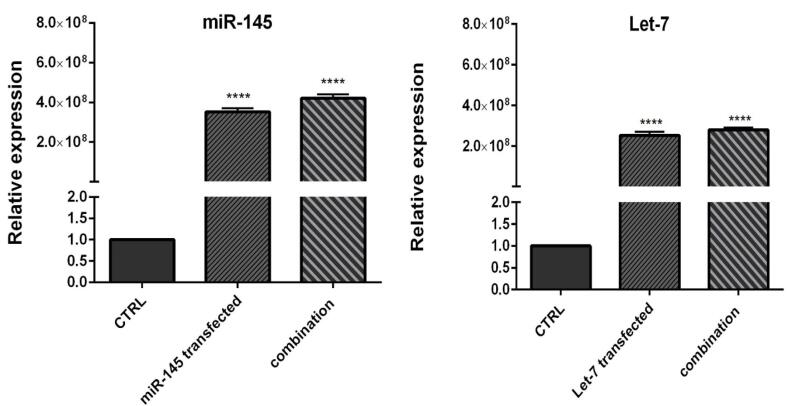
Let-7 and miR-145 expression levels in separately transfected cells and combination group compared to control. Data are indicated as the mean ± standard deviation of experiments (triplicate); ns = nonsignificant, (* *P* < 0.05), (** *P* < 0.01) and (**** *P* < 0.0001)

 To evaluate the efficiency of miRNA transfection, cells were transfected with NC miRNA labeled with FITC and analyzed with flow cytometry. The results showed that 80.5% of FITC-conjugated miRNAs were successfully transfected into HCT-116 cells (Figure 2). Furthermore, to evaluate the optimum doses and time for transfection, we used qRT-PCR technique. As illustrated in Figure 3, results showed that remarkably higher expression levels of miRNAs were observed at transfection of mimics in the concentration of 20 pmol. This overexpression was stable till 48h. After that, 20 pmol was determined as the optimal dose for transfection during 48 hours in all following experiments. Besides, significant (*P* < 0.0001) overexpression of hsa-let-7a and MIR-145 was confirmed in the combination group compared to the control (Figure 4).

###  Effect of MIR-145 and hsa-let-7a exogenous overexpression on HCT-116 cell proliferation

**Figure 5:**
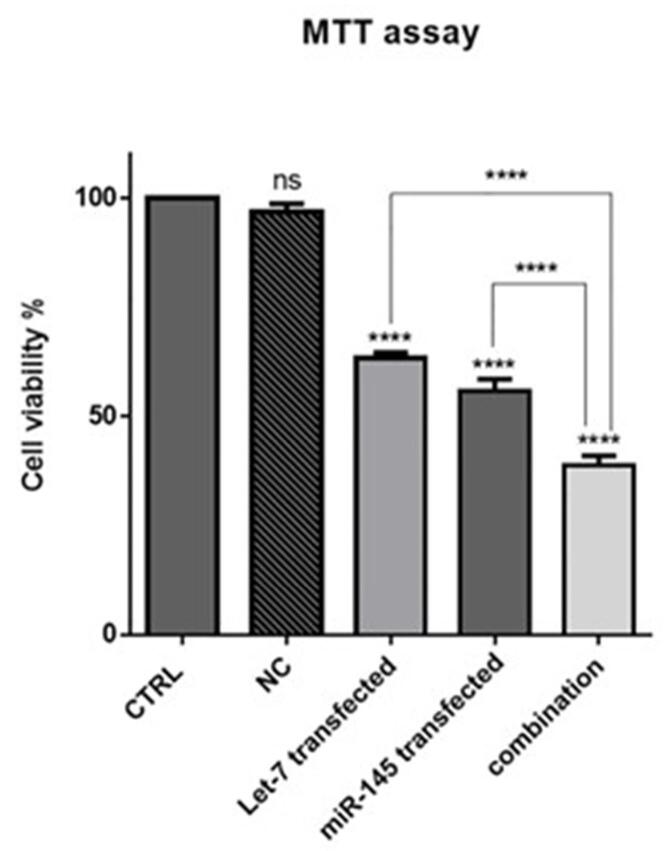
The effect of combination therapy on cell viability. MTT assay was used to evaluate cell survival after transfection of Let-7a and miR-145 into HCT-116 cell, separately and in combination; (****P < 0.0001)

 MTT assay was used to evaluate the effect of the hsa-let-7a and MIR-145 combination on cell viability and proliferation. The results showed that hsa-let-7a and MIR-145 separately could significantly decrease HCT-116 cell viability compared to control and NC groups. As illustrated in Figure 5, no significant difference was observed between the cells without transfection and cells transfected by miR-control. However, combining two miRNAs significantly reduced cell viability more effectively than individual treatments. These results are consistent with previous research findings. Marques et al reported the overexpression of hsa-let-7a in papillary thyroid carcinoma cells markedly inhibited cell growth and proliferation.^28^ Zhou et al showed that the expression of MIR-145 was downregulated in gastric cancer (GC) cells. The transfection of GC cells with MIR-145 could significantly suppress the proliferation, and migration of cancer cells.^29^

###  hsa-let-7a and MIR-145 combination increased apoptosis induction in HCT-116 cells 

**Figure 6:**
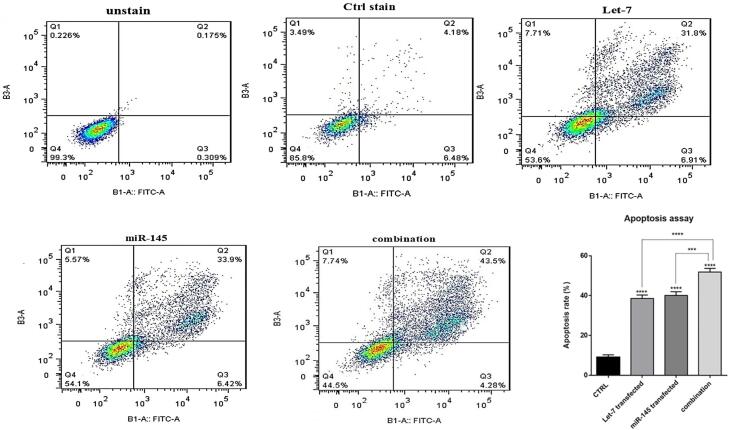
The effect of combination therapy on apoptosis. The fraction of apoptotic cells was analyzed using the V‐FITC/PI assay in HCT-116 cells under different treatments. The fractions of apoptotic cells were quantified and presented as a graph. The results were expressed as the mean ± standard deviation of experiments (repeated three times); (****P < 0.0001)

**Figure 7:**
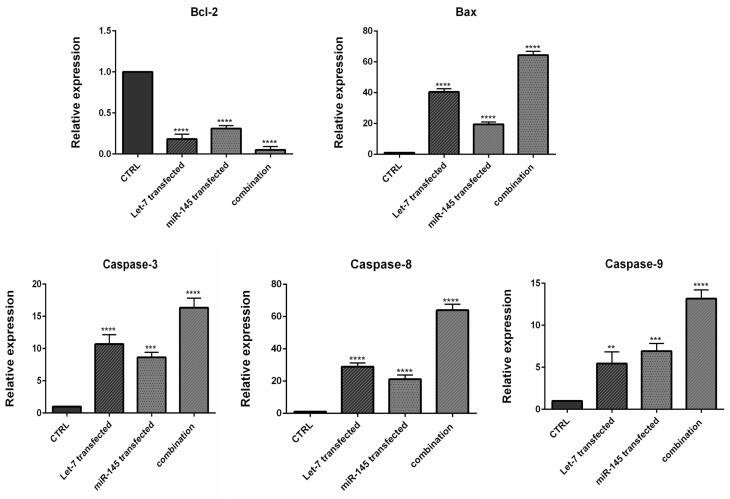
Analysis of *Bax*, *Bcl-2, Caspase ‐ 3, Caspase-8* and *Caspase ‐ 9* mRNA expression levels by qRT-PCR. The results were expressed as the mean ± standard deviation of experiments (*P < 0.05), (**P < 0.01), (***P < 0.001), and (****P < 0.0001)

 To investigate how combination therapy exerts its cytotoxic effect, flow cytometry analysis was conducted using Annexin V/PI staining. Our results showed an increased rate of apoptosis induction in HCT-116 cells transfected with mimic hsa-let-7a and mimic MIR-145 separately and simultaneously, compared to control cells, as shown in Figure 6. According to the results, the transfection of hsa-let-7a and MIR-145 individually could significantly induce apoptosis in HCT-116 cells compared to the control (*P <*0.0001). However, the combination of two miRNAs was able to significantly increase apoptosis induction more effectively than individual treatments (*P <*0.0001). To evaluate the underlying mechanism through apoptosis induction, using qRT-PCR, we quantified the expression levels of the main modulators of apoptosis. According to the results (Figure 7), significant overexpression of the pro-apoptosis genes, including *Caspase ‐ 3/8/9*,and *Bax*, was observed after transfection hsa-let-7a and MIR-145 separately in HCT-116 cells compared to the control. However, combination therapy of two miRNAs upregulated the gene expression more effectively than individual treatments. According to the results of qRT-PCR, MIR-145 and hsa-let-7a cooperatively led to significant downregulation of *Bcl-2* as an important pro-survival gene within the treated groups compared to the control group. More decreased levels of *Bcl-2* were achieved through combination therapy.

 The Caspase family plays a significant function in the regulation of cell programmed death. Between caspases, Caspase-3 is known to be the most common executioner caspase during apoptosis. Furthermore, Caspase-8 and Caspase-9 are the main initiator Caspases of the intrinsic and extrinsic pathways of apoptosis, respectively.^30^ Therefore, it could be suggested that MIR-145 and hsa-let-7a could simultaneously upregulate apoptosis pathways in CRC cells and subsequently increase cell death. let-7 microRNA family was previously reported to inhibit the expression of *Bcl-xL* gene and potentiate apoptosis induction in human hepatocellular carcinoma.^31^ Pan et al showed that increasing the expression of MIR-145 leads to *Bax* upregulation and eventually induces apoptosis in lung cancer cells.^32^ Furthermore, MIR-145 was also shown significantly induces apoptosis by modulating multiple cell death pathways.^33^ Previous studies have further shown that MIR-145 overexpression leads to upregulation of pro-apoptotic proteins like *Caspase-3*, *Caspase-9* in glioma cells and subsequently induces caspase-dependent cell death.^32,34^

###  MIR-145 and hsa-let-7a cooperatively inhibited the expression of migration regulators

**Figure 8:**
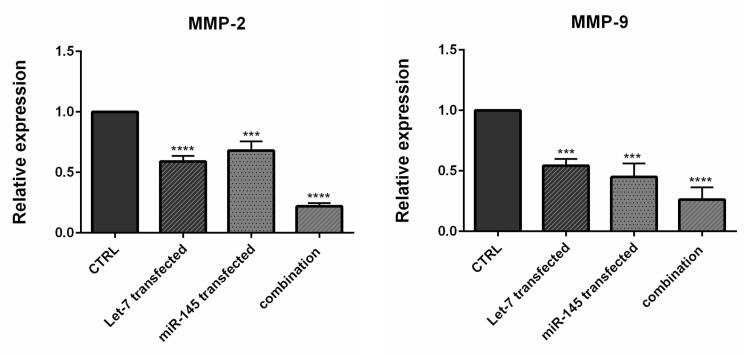
qRT-PCR showed that *MMP ‐ 2* and *MMP-9* were downregulated in transfected cells compared to controls. The results were expressed as mean ± standard deviation (triplicated); (*P < 0.05), (**P < 0.01), (***P < 0.001), and (****P < 0.0001)

 Studies have illustrated that MIR-145 and hsa-let-7a could inhibit cell migration and invasion in various cancer cell lines by modulating multiple genes.^35,36^ Using qRT-PCR, we conducted additional investigations to explore the impact of combination therapy on inhibiting genes that regulate the migration of CRC cells. We found that MIR-145 and hsa-let-7a could be important modulators of metastasis-related genes *MMP-2* and *MMP-9* in CRC cells. As shown in Figure 8, the transfection of cells individually with MIR-145 and hsa-let-7a could significantly reduce the expression levels of *MMP-2/9* compared to the control. However, the lowest expression levels of these migratory genes were observed through combination therapy, suggesting that MIR-145 and hsa-let-7a might cooperatively suppress CRC cell migration via the downregulation of these metastasis-related genes.

 Among the various proteins involved in the degradation of the extracellular matrix, MMPs, specifically MMP-9, play a significant role. This degradation process is crucial in tumor invasion, progression, and metastasis, particularly in CRC. MMP-9 has been proposed as a marker for assessing the advancement of this form of cancer.^37,38^ Epithelial-mesenchymal transition (EMT) is identified as a crucial step in the induction of CRC metastasis and invasion.^39^ During this biological process, epithelial cells undergo a transition where they acquire mesenchymal characteristics, enabling them to invade and migrate to other organs.^40^ A previous study illustrated that let-7 silencing increased expression levels of *MMP-2* and *MMP-9* and promoted the proliferation, migration, and invasion of extravillous trophoblast cells.^41^ Pan et al showed that MIR‐145 upregulation potentially triggered apoptosis of A549 cells (non-small cell lung cancer) by decreasing the expression of *MMP‐2* and *MMP‐9*.^32^ Wu et al found that let-7 imitations implicitly decreased the expression of *MMP-2*, *MMP-9*, and other EMT markers.^36^

###  hsa-let-7a and MIR-145 combination induced cell cycle arrest at the sub-G1 phase

**Figure 9:**
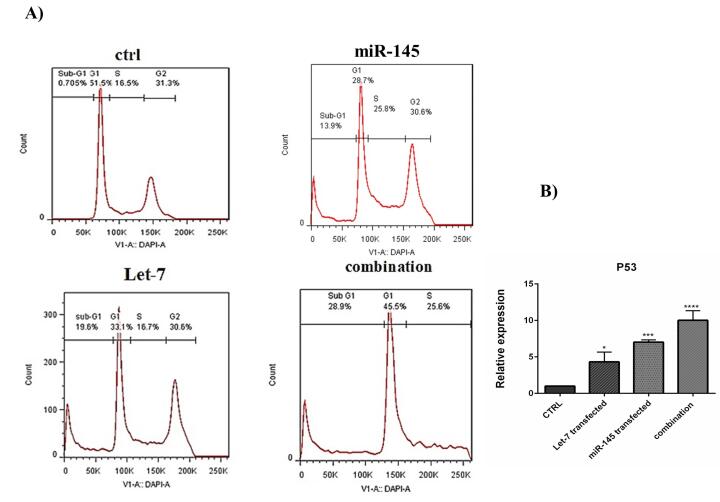
(A) The effect of combination therapy on the cell cycle was analyzed by flow cytometry. (B) expression level of *P53* (***P< 0.001)

 After transfection, cell cycle inhibition in different treatment groups was evaluated by flow cytometry. Results revealed that overexpression of hsa-let-7a and MIR-145 separately could arrest the cell cycle at the sub-G1 phase in HCT-116 cells. MIR-145 increased from 0.705% to 13.9% in the sub-G1 phase of the cell cycle. In cells treated with hsa-let-7a, the population of the sub-G1 cells also increased from 0.705% to 19.6%. However, the combination of MIR-145 and hsa-let-7a increased cell cycle arrest at the sub-G1 phase more than separate treatments to 28.9% (Figure 9A); confirming apoptosis induction results. To validate the results of the cell cycle assay, the expression level of *P53*, which is a related gene to the cell cycle, was evaluated by qRT-PCR. Our results illustrated that transfection of cells with hsa-let-7a and MIR-145 could considerably enhance the expression level of *P53*. This increase was significantly more in the combination group than individual groups (*P* < 0.001) (Figure 9B).

 These data suggested that hsa-let-7a and MIR-145 may act as regulators cell cycle and could cooperatively induce cell cycle arrest at the sub-G1 phase, confirming apoptosis induction results. Wang et al demonstrated that in breast cancer cells, overexpression of MIR-145 increased cell cycle arrest at the sub-G1 phase.^42^ Samadi et al reported that transfection of CRC cells with let-7 increased the cell arrest at sub-G1 and enhanced the radiosensitivity of cancer cells by targeting IGF‐1R.^43^ The p53 protein is known as a key player in cell cycle arrest “p53 is a transcription factor for p21 gene” and apoptosis inducer “p53 activates Bax and blocks antiapoptotic function of Bcl2 and Bcl-xL on the mitochondria.^44^ Shi et al reported that in cervical cancer cells, MIR-145 enhanced the effects of *P53* via suppressing the inhibitors of *P53*, and they showed that MIR-145 plays an essential role in *P53* tumor suppression.^45^ A study indicated that P53 induces the tristetraprolin expression in cancer cells. Tristetraprolin, in turn, increased the expression level of let-7 through the down-regulation of Lin28a. So, Tristetraprolin provides an important link between *P53* activation and let-7 biogenesis.^46^

## Conclusion

 Since conventional cancer treatments have many side effects, finding better and more effective treatments with fewer side effects is necessary in the medical and biological world today. Given the previous knowledge that miRNAs are involved in cancer suppression, their dysregulation leads to increased cancer cell proliferation. However, many miRNAs have been studied to treat cancer; so far, studies have been conducted on the association between miRNAs and different genes involved in the development and spread of cancer. Targeting these modified miRNAs in different ways has opened up new horizons for tumor inhibition and cancer treatment. In conclusion, given the specific expression pattern of MIR-145-5p and hsa-let7a-3p in CRC and their involvement in tumor progression through different signaling pathways, it may be possible in the future to serve as a molecular target for diagnostic and therapeutic aims. Due to study limitations, we did not use siRNAs, antibodies or other specialized assays, so further research is needed to confirm our conclusions through clinical studies and in vivo experiments.

## Competing Interests

 None.

## Ethical Approval

 Not applicable.

## Funding

 This study was founded by the Immunology Research Center, Tabriz University of Medical Science (Grant no. 63837).

## References

[1] Xu Y, Zhang X, Hu X, Zhou W, Zhang P, Zhang J (2018). The effects of lncRNA MALAT1 on proliferation, invasion and migration in colorectal cancer through regulating SOX9. Mol Med.

[2] Ferlay J, Colombet M, Soerjomataram I, Mathers C, Parkin DM, Piñeros M (2019). Estimating the global cancer incidence and mortality in 2018: GLOBOCAN sources and methods. Int J Cancer.

[3] Siegel RL, Torre LA, Soerjomataram I, Hayes RB, Bray F, Weber TK (2019). Global patterns and trends in colorectal cancer incidence in young adults. Gut.

[4] Hong Y, Rao Y (2019). Current status of nanoscale drug delivery systems for colorectal cancer liver metastasis. Biomed Pharmacother.

[5] Bol KF, Schreibelt G, Gerritsen WR, de Vries IJ, Figdor CG (2016). Dendritic cell-based immunotherapy: state of the art and beyond. Clin Cancer Res.

[6] Sun R, Chen P, Li L, Sun H, Nie X, Liang Y (2016). A polymorphism rs4705341 in the flanking region of miR-143/145 predicts risk and prognosis of colorectal cancer. Oncotarget.

[7] Hejazi M, Baghbani E, Amini M, Rezaei T, Aghanejad A, Mosafer J (2020). microRNA-193a and taxol combination: a new strategy for treatment of colorectal cancer. J Cell Biochem.

[8] Wang W, Ji G, Xiao X, Chen X, Qin WW, Yang F (2016). Epigenetically regulated miR-145 suppresses colon cancer invasion and metastasis by targeting LASP1. Oncotarget.

[9] Lizarbe MA, Calle-Espinosa J, Fernández-Lizarbe E, Fernández-Lizarbe S, Robles M, Olmo N (2017). Colorectal cancer: from the genetic model to posttranscriptional regulation by noncoding RNAs. Biomed Res Int.

[10] Jebelli A, Oroojalian F, Fathi F, Mokhtarzadeh A, Guardia M (2020). Recent advances in surface plasmon resonance biosensors for microRNAs detection. BiosensBioelectron.

[11] Ritterhouse LL, Samowitz WS. Genomic applications in colorectal carcinomas. In: Netto GJ, Kaul KL, eds. Genomic Applications in Pathology. Cham: Springer International Publishing; 2019. p. 393-9. 10.1007/978-3-319-96830-8_28.

[12] Treiber T, Treiber N, Meister G (2019). Regulation of microRNA biogenesis and its crosstalk with other cellular pathways. Nat Rev Mol Cell Biol.

[13] Roshani Asl E, Amini M, Najafi S, Mansoori B, Mokhtarzadeh A, Mohammadi A (2021). Interplay between MAPK/ERK signaling pathway and microRNAs: a crucial mechanism regulating cancer cell metabolism and tumor progression. Life Sci.

[14] Rezaei T, Amini M, Hashemi ZS, Mansoori B, Rezaei S, Karami H (2020). microRNA-181 serves as a dual-role regulator in the development of human cancers. Free Radic Biol Med.

[15] Domańska-Senderowska D, Laguette MN, Jegier A, Cięszczyk P, September AV, Brzeziańska-Lasota E (2019). microRNA profile and adaptive response to exercise training: a review. Int J Sports Med.

[16] Shabaninejad Z, Yousefi F, Movahedpour A, Ghasemi Y, Dokanehiifard S, Rezaei S (2019). Electrochemical-based biosensors for microRNA detection: nanotechnology comes into view. Anal Biochem.

[17] Dai X, Kaushik AC, Zhang J (2019). The emerging role of major regulatory RNAs in cancer control. Front Oncol.

[18] Asadzadeh Z, Mansoori B, Mohammadi A, Aghajani M, Haji-Asgarzadeh K, Safarzadeh E (2019). microRNAs in cancer stem cells: biology, pathways, and therapeutic opportunities. J Cell Physiol.

[19] Shen X, Jiang H, Chen Z, Lu B, Zhu Y, Mao J (2019). microRNA-145 inhibits cell migration and invasion in colorectal cancer by targeting TWIST. Onco Targets Ther.

[20] Zeinali T, Mansoori B, Mohammadi A, Baradaran B (2019). Regulatory mechanisms of miR-145 expression and the importance of its function in cancer metastasis. Biomed Pharmacother.

[21] Sadeghiyeh N, Sehati N, Mansoori B, Mohammadi A, Shanehbandi D, Khaze V (2019). microRNA-145 replacement effect on growth and migration inhibition in lung cancer cell line. Biomed Pharmacother.

[22] Xu Q, Liu LZ, Qian X, Chen Q, Jiang Y, Li D (2012). miR-145 directly targets p70S6K1 in cancer cells to inhibit tumor growth and angiogenesis. Nucleic Acids Res.

[23] Ye D, Shen Z, Zhou S (2019). Function of microRNA-145 and mechanisms underlying its role in malignant tumor diagnosis and treatment. Cancer Manag Res.

[24] Feng Y, Zhu J, Ou C, Deng Z, Chen M, Huang W (2014). microRNA-145 inhibits tumour growth and metastasis in colorectal cancer by targeting fascin-1. Br J Cancer.

[25] Li S, Wu X, Xu Y, Wu S, Li Z, Chen R (2016). miR-145 suppresses colorectal cancer cell migration and invasion by targeting an ETS-related gene. Oncol Rep.

[26] Chirshev E, Oberg KC, Ioffe YJ, Unternaehrer JJ (2019). Let-7 as biomarker, prognostic indicator, and therapy for precision medicine in cancer. Clin Transl Med.

[27] Mizuno R, Kawada K, Sakai Y (2018). The molecular basis and therapeutic potential of let-7 microRNAs against colorectal cancer. Can J Gastroenterol Hepatol.

[28] Ricarte-Filho JC, Fuziwara CS, Yamashita AS, Rezende E, da-Silva MJ, Kimura ET (2009). Effects of let-7 microRNA on Cell Growth and Differentiation of Papillary Thyroid Cancer. Transl Oncol.

[29] Zhou K, Song B, Wei M, Fang J, Xu Y (2020). miR-145-5p suppresses the proliferation, migration and invasion of gastric cancer epithelial cells via the ANGPT2/NOD_LIKE_RECEPTOR axis. Cancer Cell Int.

[30] Wu Y, Zhao D, Zhuang J, Zhang F, Xu C (2016). Caspase-8 and caspase-9 functioned differently at different stages of the cyclic stretch-induced apoptosis in human periodontal ligament cells. PLoS One.

[31] Shimizu S, Takehara T, Hikita H, Kodama T, Miyagi T, Hosui A (2010). The let-7 family of microRNAs inhibits Bcl-xL expression and potentiates sorafenib-induced apoptosis in human hepatocellular carcinoma. J Hepatol.

[32] Pan Y, Ye C, Tian Q, Yan S, Zeng X, Xiao C (2018). miR-145 suppresses the proliferation, invasion and migration of NSCLC cells by regulating the BAX/BCL-2 ratio and the caspase-3 cascade. Oncol Lett.

[33] Ostenfeld MS, Bramsen JB, Lamy P, Villadsen SB, Fristrup N, Sørensen KD (2010). miR-145 induces caspase-dependent and -independent cell death in urothelial cancer cell lines with targeting of an expression signature present in Ta bladder tumors. Oncogene.

[34] Rani SB, Rathod SS, Karthik S, Kaur N, Muzumdar D, Shiras AS (2013). miR-145 functions as a tumor-suppressive RNA by targeting Sox9 and adducin 3 in human glioma cells. Neuro Oncol.

[35] Peng X, Guo W, Liu T, Wang X, Tu X, Xiong D (2011). Identification of miRs-143 and -145 that is associated with bone metastasis of prostate cancer and involved in the regulation of EMT. PLoS One.

[36] Wu A, Wu K, Li J, Mo Y, Lin Y, Wang Y (2015). Let-7a inhibits migration, invasion and epithelial-mesenchymal transition by targeting HMGA2 in nasopharyngeal carcinoma. J Transl Med.

[37] Zeng ZS, Guillem JG (1995). Distinct pattern of matrix metalloproteinase 9 and tissue inhibitor of metalloproteinase 1 mRNA expression in human colorectal cancer and liver metastases. Br J Cancer.

[38] Herszényi L, Hritz I, Lakatos G, Varga MZ, Tulassay Z (2012). The behavior of matrix metalloproteinases and their inhibitors in colorectal cancer. Int J Mol Sci.

[39] Natalwala A, Spychal R, Tselepis C (2008). Epithelial-mesenchymal transition mediated tumourigenesis in the gastrointestinal tract. World J Gastroenterol.

[40] Xu T, Jing C, Shi Y, Miao R, Peng L, Kong S (2015). microRNA-20a enhances the epithelial-to-mesenchymal transition of colorectal cancer cells by modulating matrix metalloproteinases. Exp Ther Med.

[41] Zhang L, Wang K, Wu Q, Jin L, Lu H, Shi Y (2019). Let-7 inhibits the migration and invasion of extravillous trophoblast cell via targeting MDM4. Mol Cell Probes.

[42] Wang S, Bian C, Yang Z, Bo Y, Li J, Zeng L (2009). miR-145 inhibits breast cancer cell growth through RTKN. Int J Oncol.

[43] Samadi P, Afshar S, Amini R, Najafi R, Mahdavinezhad A, Sedighi Pashaki A (2019). Let-7e enhances the radiosensitivity of colorectal cancer cells by directly targeting insulin-like growth factor 1 receptor. J Cell Physiol.

[44] Jahanafrooz Z, Motamed N, Rinner B, Mokhtarzadeh A, Baradaran B (2018). Silibinin to improve cancer therapeutic, as an apoptotic inducer, autophagy modulator, cell cycle inhibitor, and microRNAs regulator. Life Sci.

[45] Shi M, Du L, Liu D, Qian L, Hu M, Yu M (2012). Glucocorticoid regulation of a novel HPV-E6-p53-miR-145 pathway modulates invasion and therapy resistance of cervical cancer cells. J Pathol.

[46] Lee JY, Kim HJ, Yoon NA, Lee WH, Min YJ, Ko BK (2013). Tumor suppressor p53 plays a key role in induction of both tristetraprolin and let-7 in human cancer cells. Nucleic Acids Res.

